# Parental beliefs about portion size, not children's own beliefs, predict child BMI

**DOI:** 10.1111/ijpo.12218

**Published:** 2017-04-04

**Authors:** C. Potter, D. Ferriday, R. L. Griggs, J. P. Hamilton‐Shield, P. J. Rogers, J. M. Brunstrom

**Affiliations:** ^1^ Nutrition and Behaviour Unit, School of Experimental Psychology University of Bristol Bristol UK; ^2^ NIHR Biomedical Research Unit in Nutrition University of Bristol Bristol UK

**Keywords:** BMI, children, eating behaviour, obesity, parental feeding practices, portion size

## Abstract

**Background:**

Increases in portion size are thought by many to promote obesity in children. However, this relationship remains unclear. Here, we explore the extent to which a child's BMI is predicted both by parental beliefs about their child's ideal and maximum portion size and/or by the child's own beliefs.

**Methods:**

Parent–child (5–11 years) dyads (*N* = 217) were recruited from a randomized controlled trial (*n* = 69) and an interactive science centre (*n* = 148). For a range of main meals, parents estimated their child's ‘ideal’ and ‘maximum tolerated’ portions. Children completed the same tasks.

**Results:**

An association was found between parents' beliefs about their child's ideal (*β* = .34, *p* < .001) and maximum tolerated (*β* = .30, *p* < .001) portions, and their child's BMI. By contrast, children's self‐reported ideal (*β* = .02, *p =* .718) and maximum tolerated (*β* = −.09, *p* = .214) portions did not predict their BMI. With increasing child BMI, parents' estimations aligned more closely with their child's own selected portions.

**Conclusions:**

Our findings suggest that when a parent selects a smaller portion for their child than their child self‐selects, then the child is less likely to be obese. Therefore, public health measures to prevent obesity might include instructions to parents on appropriate portions for young children.

## Introduction

Despite being regarded as a serious global health concern, rates of childhood obesity continue to increase 1, 2. This is a concern because obesity in childhood greatly increases the risk of being overweight in adulthood 2. Recently, food portion sizes have also increased 3, and several researchers suggest that larger portions have promoted obesity 4, 5. Consistent with this hypothesis, both adults 6 and children 7 consume significantly more food when presented with larger portions. However, notably, there is relatively little evidence 8 that overweight individuals actually select larger portions, and this relationship remains unclear. Some have found BMI to be a positive predictor of portion size selection 9, 10, whereas others have found no relationship 11. To our knowledge, only one study has assessed the relationship between self‐selected portion size and BMI in young children (aged 3–5 years) 12 – those children who self‐selected larger portions tended to be overweight.

The selection of larger portions may reflect a greater tolerance for large portions. Obese adults may have a relatively larger gastric capacity 13. However, the extent to which this corresponds with the selection of larger portions remains unclear. To date, the relationship between children's tolerance of (and willingness to accept) large portions and child BMI has not been explored. Parents tend to select their child's portions at mealtimes 14 and often encourage their children to consume all of food that they are served 15. However, it has been suggested that parents lack an understanding of age‐appropriate portions for their children 16. Perhaps, for this reason, portion size plays an important role in determining energy intake in children 17.

In the present study, as a primary aim, we sought to determine the relative importance of (i) parental beliefs about their child's ideal and maximum tolerated portion sizes and (ii) the child's own portion size preferences, as predictors of child BMI percentile. In this context, we were also interested to identify sources that shape portion selections made by both children and by parents for their children. Several studies indicate that children model their dietary behaviour on their parents' behaviour 18, 19. Therefore, as a secondary aim, we hypothesized that children's own portion preferences might mirror those of their parents and that parental portion estimates for their children might be motivated by trends in their own self‐selected portions 14. To assess these relationships, we asked parents to report their own ideal portions.

## Methods

### Subjects

Child–parent (or primary caregiver) dyads (*N* = 217) were recruited from two sources: (i) overweight children (BMI > 95th percentile) who were referred to a National Institute of Health Research (NIHR) randomized trial (Health Technology Assessment (HTA) Ref 18470, *n* = 69) 20 and (ii) an opportunity sample of overweight and lean children from a local interactive science centre (*n* = 148) in Bristol (UK). All children were English‐speaking and aged between 5 and 11 years. Children with food allergies or intolerances were excluded, together with vegetarians and vegans. After additional data cleaning (see Supporting Information *Note S1*), 203 child–parent (or primary caregiver) dyads remained. The study protocol was approved through both the National Health Service HTA Programme (ref: 09/127/04) and the local Human Research Ethics Committee. Financial compensation was not provided.

### Food images

Our food images comprised seven main meals. We selected foods that were likely to be familiar and well liked by children in the local area. The main meals consisted of (i) chicken, chips (fries) and baked beans; (ii) chicken curry with rice; (iii) spaghetti Bolognese; (iv) lasagne and peas; (v) macaroni and cheese; (vi) sausage, mashed potatoes and peas; and (vii) pizza and chips (fries). Each meal was photographed 50 times (numbered 1–50) on the same white plate (255‐mm diameter). Across the range of pictures, the portion sizes increased in equicaloric 20‐kcal steps and started with a 20‐kcal portion. See Table S1 for the energy density and macronutrient composition of the meals.

### Measures

#### Ideal portion size

We assessed parents' and children's ideal portion sizes using methods that have been validated elsewhere 21. An image (150‐mm × 150‐mm) was displayed on a 17.3″ TFT‐LCD laptop. First, parents and children were asked to ‘Imagine you are going to eat this food for dinner and no other food is available. What would be your perfect amount for dinner?’ Parents were then asked to estimate their child's ideal portion size. They were instructed to ‘Imagine your child is going to eat this food for dinner and no other food is available. What would be your child's perfect amount for dinner?’ All instructions were read aloud to participants.

Participants changed the portion size of each meal using the left and right arrow keys on the keyboard. Depressing the left arrow caused the portion size to decrease and depressing the right arrow caused the portion size to increase. For each participant, the order of the meals was randomized, as was the initial portion size in each trial. For each child, we calculated a mean ‘ideal portion‐size’ (kcal) by averaging responses across foods. The ability for younger children to engage with this task is considered in the Discussion.

#### Maximum portion size

Children were asked to ‘Imagine you are going to eat this food for dinner and no other food is available. What is the most you could eat for dinner?’ Parents were asked to provide portion estimates in response to the instruction ‘Imagine your child is going to eat this food for dinner and no other food is available. What would be the most that your child could eat for dinner?’ In all other respects, the methods were identical to those in the ideal portion‐size task.

#### Familiarity

Meal images (400‐kcal portions) were displayed on the laptop screen in a randomized order. Parents were asked ‘How often does your child eat this food?’ and were instructed to select one of the following options: (i) ‘Never or rarely’; (ii) ‘About once every two months’; (iii) ‘About once every two weeks’; (iv) ‘One or two times a week’; or (v) ‘Most days.’

#### Liking

Meal images (400‐kcal portions) were presented in succession on the laptop screen in a randomized order. Children used a computerized 100‐mm visual‐analogue rating scale headed ‘How much do you LIKE the taste of this food?’ with anchor points ‘I hate it’ to ‘I love it’ to rate their level of liking for each food. To determine whether children's portion size selection was modified by their liking for the test foods, we calculated an average liking score across familiar foods (see Supporting Information *Note S1*).

### Procedure

Parents read an information sheet before providing written consent. This also incorporated consent for their child. Participants in the NIHR trial completed the study visit in their homes. Participants from the science centre were tested in a private booth. Parents and children sat at opposite ends of a table, at separate laptops, where they were unable to view each other's responses. Prior to completing the portion size tasks, participants were shown a physical plate that was identical to the plate displayed in the portion size tasks. Children completed tasks in the following order: (i) own ideal portion size; (ii) own maximum portion size; and (iii) liking. Parents completed tasks in the following order: (i) own ideal portion size; (ii) child's ideal portion size; (iii) child's maximum portion size; and (iv) familiarity. Parents then reported their marital status, their current employment status and their child's date of birth. All participants' height was then measured to the nearest millimetre using a stadiometer. Their weight was measured to the nearest 0.1 kg using a Tanita TBF‐531 digital scale. Finally, parents were given a debriefing sheet which explained the broad aims of the research. Each session lasted approximately 15 min.

### Data analysis

To take into account age and sex differences, standardized child BMI percentiles were computed using a BMI percentile calculator for children and teens 22. In the first instance, all raw data were inspected and screened (see Supporting Information *Note S1* for further details). To evaluate their independent contribution as predictors of child BMI percentile, we entered the following variables simultaneously in a linear regression model: children's self‐selected ideal portion size, children's self‐selected maximum portion size, liking, parent's own ideal portion size, parent estimates of children's ideal portions, parent estimates of children's maximum portions and parent BMI. However, the resulting model was distorted by multicollinearity; the average variance inflation factor (VIF) was 1.66, and a value above one is generally considered an indicator of multicollinearity 23 (see Table 1 for correlations between these variables). Inspection of the data suggested that the primary source of multicollinearity was the significant relationship between parent's estimates of their child's ideal portion size (VIF = 2.73) and parent's estimates of their child's maximum portion size (VIF = 2.77) (*r* (196) = .78, *p* < .001).

**Table 1:** Pearson's correlations (*r*) to assess relationships between variables

	Parent BMI	Child IP	Child MP	Parent IP	Parent IP estimate	Parent MP estimate	Liking
Child BMI (%)	0.33***	0.13*	0.006	−0.07	0.39***	0.30***	0.10
Parent BMI	—	0.14*	0.026	0.07	0.22***	0.14*	0.09
Child IP		—	0.543***	−0.07	0.15*	0.16*	0.10
Child MP			—	−0.03	0.2**	0.22***	0.07
Parent IP				—	0.06	0.20**	0.06
Parent IP estimate					—	0.78***	0.08
Parent MP estimate						—	0.05

*Notes*. IP, ideal portion size; MP, maximum portion size; IP estimate, parent estimates of their child's ideal portion size; MP estimate, parent estimates of their child's maximum portion size.

For all variables, *n* = 198.

* *p* < .05.

** *p* < .01.

*** *p* < .001.

In response to this concern, we performed separate analyses on data relating to ideal and maximum portion sizes. In one regression model, we entered children's self‐reported ideal portion size, liking, parental estimates of their child's ideal portion size, parent BMI and the parents' own ideal portion size as independent predictors of child BMI percentile. In a second analysis, we entered children's self‐reported maximum portion size, liking, parental estimates of the child's maximum portion size and parent BMI. Statistical analyses were performed using SPSS version 21.0.0 (SPSS Inc., Chicago, IL, USA). Finally, we note that all of these analyses were also conducted on the full data set (before excluding any participants or values). Further, to take into account potential ‘test site effects’, we ran the same analyses including only data from children from the science centre because this sample contained both lean and overweight children. In every case, the same pattern of findings was preserved, and all significant and non‐significant results remained unchanged.

## Results

### Demographics

Our final sample had a mean age of 8.1 years (*SD*: 1.8, range: 5–11). Child BMI percentiles ranged from 1 to 99% (mean = 73.1%, *SD*: 27.4), and parent BMI ranged from 17.2 to 51.8 kg/m^2^ (mean = 28.0, *SD*: 6.2). Roughly half of the children were lean (BMI < 85^th^ percentile, *n =* 110), and half were overweight (BMI > 85^th^ percentile, *n* = 101). Notably, nearly a third (*n* = 32) of the overweight children were recruited from the interactive science centre. A slightly higher proportion of parents were overweight (BMI ≥ 25 kg/m^2^, *n* = 122) than lean (BMI < 25 kg/m^2^ *, n* = 83). *Table S2* shows the distribution of children by weight status and gender. The majority of parents were married or living with a partner (76% of lean parents and 79% of overweight parents), and most were employed in either full‐ or part‐time work (74% of lean parents and 84% of overweight parents).

### Predictors of BMI in 5‐ to 11‐year‐old children

The first regression model (Table 2 *)* accounted for 24% of the variance in child BMI percentile. Parent BMI and parent estimates of their child's ideal portion size positively predicted child BMI percentile. By contrast, children's own ideal portion size, liking for meals and parents' own ideal portion size did not predict child BMI percentile.

**Table 2:** Standard multiple regression of portion size variables on child BMI percentile

Variables	Mean (SD)	*B*	*β*	95% CI	*sr* ^*2*^ (unique)	Model fit
*Model 1*						
Child IP (kcal)	470 (167)	0.005	0.03	(−.016, .027)	0.001	*R* ^2^ = .24
Parent IP (kcal)	495 (137)	−0.023	−0.12	(−.049, .002)	0.013	Adjusted *R* ^2^ = .22
Parent IP est. (kcal)	395 (124)	0.074***	0.33	(.046, .103)	0.1	*R* = .49***
Parent BMI	27.9 (6.2)	1.170***	0.26	(.594, 1.745)	0.064	Intercept =14.962
Liking	55.0 (17.9)	0.076	0.05	(−.118, .271)	0.002	
*Model 2*						
Child MP (kcal)	615 (196)	−0.009	−0.07	(−.028, .009)	0.003	*R* ^2^ = .19
Parent MP est. (kcal)	508 (140)	0.054***	0.27	(.028, .080)	0.07	Adjusted *R* ^2^ = .17
Parent BMI	27.9 (6.2)	1.304***	0.29	(.725, 1.884)	0.08	*R* = .43***
Liking	55.0 (17.9)	0.096	0.06	(−.103, .295)	0.004	Intercept = 8.56

*Notes*. IP, ideal portion size; MP, maximum portion size; parent IP est., parent estimates of their child's ideal portion size; parent MP est., parent estimates of their child's maximum portion size.

*** *p <* .001.

**Figure 1:**
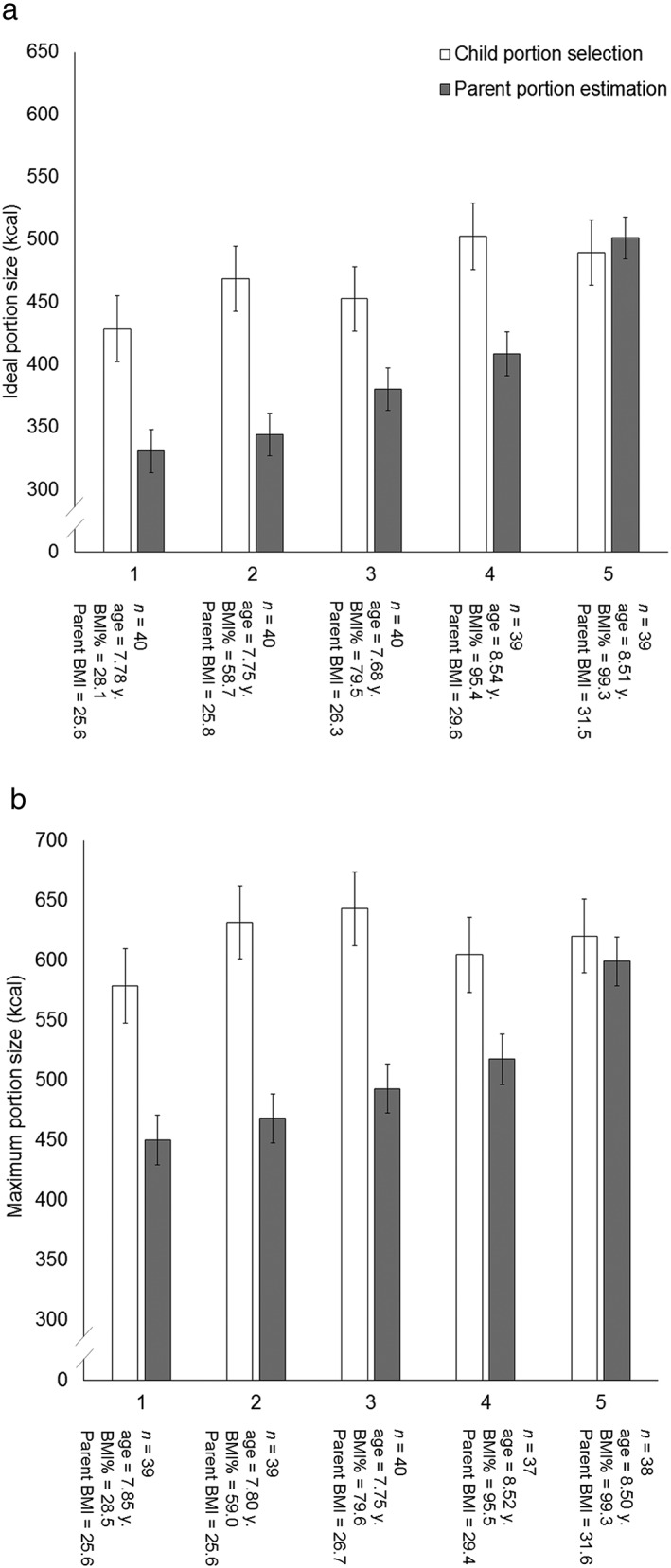
Children's self‐selected ideal (A) and maximum (B) portion sizes (kcal) and parent's estimates of their child's ideal and maximum portions (kcal), separated by quintile. The number of child–parent dyads (*n*)*,* average child age (years), child BMI percentile and parent BMI are displayed below each quintile. Quintiles are displayed in order of ascending child BMI percentile.

The second regression model (Table 2) accounted for 19% of the variance in child BMI percentile. Parent BMI and parent estimates of their child's maximum portion size positively predicted child BMI percentile. Children's self‐selected maximum portion size and liking for meals were not significant independent predictors in this model. To visually illustrate these relationships, Fig. 1 shows mean ideal (panel A) and mean maximum (panel B) portion sizes. Separate values are provided for portions selected by children and portions estimated by their parents. To highlight the magnitude of these differences, we have subdivided our sample by performing a quintile split of BMI percentiles. As shown in Fig. 1, parents of lean children (quintile 1) believed their children would select smaller portions for themselves than their children actually selected. In other words, the parents of especially lean children tended to underestimate their children's own portion size selections. With increasing child BMI quintile, parents' portion‐size estimations tended to align more closely with their child's self‐selected portions. Finally, to address our secondary aim, correlations between our predictors demonstrate that parents' estimates of their child's ideal portion were not associated with own ideal portion size (*r* = .06, *p* = ns). Further, children's self‐selected ideal portions were not correlated with their parents' ideal portion size (*r* = −.07, *p* = ns) (see Table 1).

## Discussion

Parents who believed that their child had larger ideal and maximum portions were more likely to have an overweight or obese child. By contrast, children's portion‐size selections did not predict their BMI. Moreover, this pattern was observed after accounting for variance explained by children's liking for the meals, parental BMI and parental ideal portion size. This association is in contrast to previous work 12 showing a preference for larger portions in overweight children. One possibility is that parents of an obese or overweight child ‘overfeed’ because they overestimate their child's ideal portions. However, our data (Fig. 1, Panel A) suggest the converse – portions estimated by parents of overweight children were nearly identical (1.0‐kcal difference) to those selected by their children. By contrast, portions estimated by parents of lean children were much smaller (106‐kcal smaller) than their children's own portion preferences. Therefore, another possibility is that children are protected from becoming overweight when their parents provide smaller portions. For now, we are unable to disentangle these two different accounts.

In a previous study, differences in the perceived portion size capacity between individuals with a high and low body weight were observed 13. By contrast, in this study, overweight and lean children did not differ in their reported maximum tolerated portions. However, again, we see a difference across parents' estimations of their child's portion size tolerance (Fig. 1, Panel B) – parents of obese children believed their children could tolerate larger portions than those estimated by parents of lean children (113‐kcal difference). This indicates that obese–lean differences are not limited to parents' beliefs about their child's ideal portion. Indeed, parents' beliefs about their child's physical maximum capacity might play a role in determining their beliefs about their child's ideal portion. Therefore, it might also be the case that parents' beliefs about their children's preferences are in response to their child's current BMI. For now, evidence for causation is lacking but might be explored in future studies.

To address our second hypothesis, we asked parents to report their own ideal portions. Here, as parents' own ideal portion size did not correlate with their child's ideal portion size (see Table 1), it is unlikely that children simply model their portion preferences on those of their parent. Further, we noted that parental portion estimates might be motivated by trends in their own self‐selected portions 14. Again, we failed to find evidence for this association (see Table 1).

To our knowledge, this is the first time that a computer‐based task has been used to assess portion selections in young children. A potential concern is that children lack the ability to use these computerized assessments. We take this concern seriously, because it challenges our proposition about the importance of parental portion decisions. In response, we believe it might be helpful to further deconstruct the nature of this concern. One possibility is that children are unable to use any abstract task of this kind. In this regard, we would suggest that there is ample evidence to indicate that this is not the case. For example, children as young as 4 years can make similar portion‐size assessments using computerized food images 24. If we therefore assume that children are able to perform such tasks, one of two conclusions remains. First, children can use IT to perform abstract tasks but lack the specific ability to select their own portions. Second, children can use IT to perform abstract tasks and are able to select their own portions. Either way, neither outcome would be consistent with an account based on obese–lean differences in children's portion preferences playing a role in promoting adiposity.

In conclusion, parental beliefs about their child's ideal and maximum portion sizes may play a more important role in determining their child's BMI than previously thought. Children's own portion selections tended to be high, regardless of their BMI, and are perhaps less reliable for this reason. Our data suggest that, after controlling for parent BMI, parental beliefs about their child's ideal and maximum portions account for 10.3 and 7.0% of the variance in child BMI percentile, respectively. This is not dissimilar to other well‐established predictors, such as parent BMI, which accounted for 6.4 and 8.3% of the variance in child BMI percentile in each model, respectively. One limitation to this study was that parent gender was not recorded; therefore, we cannot conclude whether parent gender influences these relationships. Additionally, several well‐documented modifiable predictors of child BMI were not assessed, such as children's level of energy expenditure and other parental feeding practices (e.g. parental control 25). However, as beliefs about portion size are modifiable, our findings may help to inform targeted public health obesity intervention programs aimed at reducing obesity in children.

## Conflict of Interest Statement

No conflict of interest was declared.

## Supporting information


**Table S1.** Energy density and macronutrient composition of test meals for ideal and maximum portion size tasks (per 100 g).
**Table S2**. Child characteristics.
