# Supplementary material for: Parental beliefs about portion size, not children's own beliefs, predict child BMI
Source: Pediatr Obes. 2017 Apr 4;13(4):232–8. doi: 10.1111/ijpo.12218 (PMC5873371; doi:10.1111/ijpo.12218)
Supplement: Supplementary file 1 — Table S1. Energy density and macronutrient composition of test meals for ideal and maximum portion size tasks (per 100 g). Table S2. Child characteristics. [file IJPO-13-232-s001.docx]

**Supporting Information**

**Parental beliefs about portion size, not children’s own beliefs,
predict child BMI**

Christina Potter^1^*, Danielle Ferriday^1^, Rebecca L. Griggs^1^, Julian P. Hamilton-Shield^2^, Peter J. Rogers^1^, Jeffrey M. Brunstrom^1^

^1^Nutrition and Behaviour Unit, School of Experimental Psychology, University of Bristol, 12a Priory Road, Bristol, BS8 1TU, UK

^2­^ NIHR Biomedical Research Unit in Nutrition, University of Bristol, Bristol, BS2 8AE, UK

*corresponding author

Christina Potter,

Nutrition and Behaviour Unit,

School of Experimental Psychology,

University of Bristol,

12a Priory Road,

Clifton, Bristol,

BS8 1TU, UK.

E-mail address: christina.potter@bristol.ac.uk

**Supporting Information**

***Note S1***

**Data screening**

One child with a learning disability struggled to complete the tasks and was excluded for this reason. We also excluded children with a vegetarian or vegan parent (*n*=5) and one child with a parent who misreported the child’s date of birth (we were unable to compute a BMI percentile). Six parents opted out of the weight measurement. *A priori* we decided that if a parent reported that their child was unfamiliar with a meal (familiarity response = ‘never or rarely’) then responses associated with that meal would be excluded from our analyses. Eight parents did not complete the familiarity measure, therefore responses to these meals (*n* = 112 meals across both portion size tasks, 3.8% of the total number of meals) were excluded from our analyses. Parents who did complete the familiarity questionnaire (96% of the sample, *N*= 203), reported that their children were generally familiar with the meals. However, one child was unfamiliar with all seven meals and their data were excluded. In cases where children were unfamiliar with a meal (18% of all responses), data associated with that meal were excluded. The least familiar foods were chicken curry with rice (39.4% of children had never had this meal) and macaroni and cheese (32.0% of children had never had this meal).

**Supporting Information**

| ***Table S1.* Energy density and macronutrient composition of test meals for ideal and maximum portion size tasks (per 100g)** | | | | | | | | | |
| --- | --- | --- | --- | --- | --- | --- | --- | --- | --- |
|  |  |  |  |  |  |  |  |  |  |
| **Main meal** | **Components** | **Grams** | **Kcal** | **Protein (g)** | **Carb (g)** | **Fat (g)** | **Fibre (g)** | **Sodium (g)** | **Energy Density** |
| **Chicken, chips and baked beans** | *Sainsbury's Breaded Chicken Escalopes* | 37 | 89 | 6.4 | 5.6 | 4.4 | 0.5 | 0.2 | **2.26** |
|  | *Sainsbury's French Fries* | 51.8 | 128 | 1.7 | 20.3 | 4 | 1.9 | 0.04 |  |
|  | *Heinz Baked Beans* | 11.25 | 9 | 0.5 | 1.45 | 0.02 | 0.4 | 0.03 |  |
|  | ***Total*** | **100** | **226** | **8.6** | **27.35** | **8.42** | **2.8** | **0.27** |  |
| **Chicken curry** | ***Sainsbury's Indian Chicken Tikka Masala & Rice*** | **100** | **168** | **9.1** | **18.1** | **6.6** | **0.1** | **0.2** | **1.68** |
| **Lasagne and peas** | *Sainsbury's Italian Beef Lasagne* | 90.8 | 138 | 6.2 | 12 | 6.7 | 2.8 | 0.2 | **1.45** |
|  | *Sainsbury's Basics British Garden Peas* | 9.3 | 7 | 0.6 | 0.8 | 0.1 | 0.5 | Trace |  |
|  | ***Total*** | **100** | **145** | **6.8** | **12.8** | **6.8** | **3.3** | **0.2** |  |
| **Macaroni and cheese** | ***Sainsbury's Italian Macaroni Cheese*** | **100** | **151** | **7** | **14** | **7.4** | **1.6** | **0.2** | **1.51** |
| **Pizza and chips** | *Chicago Town Deep Dish Pepperoni Pizza* | 58 | 173 | 6.8 | 1.7 | 7.8 | 0.9 | Trace | **2.77** |
|  | *Sainsbury's French Fries* | 42 | 104 | 1.3 | 16.5 | 3.2 | 1.6 | 0 |  |
|  | ***Total*** | **100** | **277** | **8.1** | **18.2** | **11** | **2.5** | **0** |  |
| **Sausage, mash and peas** | *Essential Waitrose British Pork Sausages* | 55.9 | 133 | 7.2 | 4 | 9.8 | 1 | 0.3 | **1.63** |
|  | *Sainsbury's Ready Mashed Maris Piper Potato* | 33.6 | 23 | 0.5 | 3.8 | 0.5 | 0.9 | Trace |  |
|  | *Sainsbury's Basics British Garden Peas* | 10.5 | 7 | 0.6 | 1 | 0.1 | 0.6 | Trace |  |
|  | ***Total*** | **100** | **163** | **8.3** | **8.7** | **10.4** | **2.5** | **0.3** |  |
| **Spaghetti Bolognese** | ***Sainsbury's Italian Spaghetti Bolognese*** | **100** | **141** | **7.2** | **16.2** | **5.3** | **2.1** | **0.1** | **1.41** |

**Supporting Information**

| ***Table S2.* Child characteristics** | | |
| --- | --- | --- |
|  | **Lean** | **Overweight** |
| **Female *(%)*** | 54.9 | 59.6 |
| **Total *(n)*** | 110 | 100 |
| **Total (% of cohort)** | 52.4 | 47.7 |
